# *In vitro* Evaluation of the Anti-inflammatory Effects of Thymoquinone in Osteoarthritis and *in silico* Analysis of Inter-Related Pathways in Age-Related Degenerative Diseases

**DOI:** 10.3389/fcell.2020.00646

**Published:** 2020-07-23

**Authors:** Gauthaman Kalamegam, Saadiah M. Alfakeeh, Afnan Omar Bahmaid, Etimad A. AlHuwait, Mamdouh A. Gari, Mohammed M. Abbas, Farid Ahmed, Muhammed Abu-Elmagd, Peter Natesan Pushparaj

**Affiliations:** ^1^Stem Cells Unit, Centre of Excellence in Genomic Medicine Research (CEGMR), King Abdulaziz University, Jeddah, Saudi Arabia; ^2^Department of Medical Laboratory Technology, Faculty of Applied Medical Sciences, King Abdulaziz University, Jeddah, Saudi Arabia; ^3^Sheikh Salem Bin Mahfouz Scientific Chair for Treatment of Osteoarthritis by Stem Cells, King Abdulaziz University, Jeddah, Saudi Arabia; ^4^Faculty of Medicine, Asian Institute of Medicine, Science and Technology, AIMST University, Bedong, Malaysia; ^5^Department of Biochemistry, Faculty of Applied Medical Sciences, King Abdulaziz University, Jeddah, Saudi Arabia; ^6^Department of Orthopaedic Surgery, Faculty of Medicine, King Abdulaziz University, Jeddah, Saudi Arabia

**Keywords:** stem cells, osteoarthritis, thymoquinone, inflammation, aging, ingenuity pathway analysis, SwissTargetPrediction

## Abstract

Chronic inflammation is a common underlying factor in osteoarthritis (OA) and most age-related degenerative diseases. As conventional therapies help only in partial alleviation of symptoms in OA, stem cell-based therapies and herbal supplements are being widely explored. Thymoquinone (TQ), an active ingredient of *Nigella sativa* is reported to have immunomodulatory, anti-inflammatory and antioxidant properties. We evaluated the effects of TQ on bone marrow MSCs (BM-MSCs) derived from OA patients and its interrelated pathways in inflammation and age-related degenerative diseases using Ingenuity Pathway Analysis (IPA) as well as possible molecular targets using SwissTargetPrediction. BM-MSCs were derived from OA patients and their stemness properties were characterized by studying the MSCs related CD surface marker expression and differentiation into adipocytes, osteoblasts, and chondrocytes. Treatment with TQ (100 nM–5 μM) demonstrated cell death, especially at higher concentrations. MTT assay demonstrated a significant concentration-dependent decrease in cell viability which ranged from 20.04% to 69.76% with higher doses (300 nM, 1 μM, and 5 μM), especially at 48h and 72h. Additional cell viability testing with CellTiter-Blue also demonstrated a significant concentration-dependent decrease in cell viability which ranged from 27.80 to 73.67% with higher doses (300 nM, 1 μM, 3 μM, and 5 μM). Gene expression analysis following treatment of BM-MSCs with TQ (1 and 3 μM) for 48h showed upregulation of the anti-inflammatory genes *IL-4* and *IL-10*. In contrast, the pro-inflammatory genes namely *IFN-*γ, *TNF-*α, *COX-2*, *IL-6*, *IL-8*, *IL-16*, and *IL-12A* although were upregulated, compared to the lower concentration of TQ (1 μM) they were all decreased at 3 μM. The pro-apoptotic *BAX* gene was downregulated while the *SURVIVIN* gene was upregulated. IPA of the molecular interaction of TQ in inflammation and age-related degenerative diseases identified canonical pathways directly related to synaptogenesis, neuroinflammation, TGF-β, and interleukin signaling. Further screening led to the identification of 36 molecules that are involved in apoptosis, cell cycle regulation, cytokines, chemokines, and growth factors. SwissTargetPrediction of TQ identified potential molecular targets with high probability. TQ exerted anti-inflammatory effects and therefore can be a useful adjuvant along with conventional therapies against inflammation in OA and other age-related degenerative diseases.

## Introduction

Physiological age-related decline in function of the various tissues in the human body is inevitable. However, chronic infections and diseases accompanying aging process of different tissues may result in pathological degeneration of the respective tissue. Age-related degenerative diseases are associated with cellular and biochemical damage contributing to both functional and structural degeneration of the organ systems (da Costa et al., 2016; Cole and Franke, 2017; Ramalingam et al., 2018). Such degenerative processes become commonly manifested as neurological diseases (Alzheimer’s and Parkinson’s) bone and joint diseases (OA, rheumatoid arthritis, and osteoporosis), retinal diseases (macular degeneration, glaucoma, cataract, and diabetic retinopathy) or cardiovascular diseases (atherosclerosis, hypertension, and valvular stenosis). Aging related chronic diseases and degenerative disorders have major economic and social implications. Therefore, they need serious interventional strategies (Tarailo-Graovac et al., 2016; Ramalingam et al., 2018).

Osteoarthritis is a degenerative disease, especially of the weight-bearing joints including knees, pelvis and lumbosacral spine. Also, the joints that are most commonly used such as the interphalangeal and metacarpal joints are prone to develop OA. Although OA was once considered to be a non-inflammatory arthropathy, current knowledge indicates the presence of underlying chronic inflammation. Aging, obesity, traumatic injuries, and genetic causes are some of the major risk factors which predispose to the development of OA (Wang et al., 2011). The underlying pathology in OA is the articular cartilage damage associated with inflammation, swelling of the joint, pain, stiffness, loss of mobility and long-term incapacitation due to poor intrinsic healing ability (Mobasheri et al., 2014; Kalamegam et al., 2018a). Conventional therapy includes modification of lifestyle factors, exercises, analgesics, cyclo-oxygenase inhibitors especially in early OA with partial cartilage damage (Ondresik et al., 2017), and surgical management including microfracture (Pridie drilling), mosaicplasty or total knee replacement in chronic OA with full-thickness cartilage destruction (Quinn et al., 2018).

Studies using animal models have provided a great understanding of the cellular and molecular changes that prevails in OA. Dysregulation of the TGF-β superfamily, Notch, Indian hedgehog, and Wnt/β-catenin signaling pathways become involved in induction and activation of catabolic events leading to development and progression of OA (Lin et al., 2009; Sassi et al., 2014). Importantly, ‘inflammaging’ which is a state of low-grade inflammation with an upregulated immune response is understood to be the underlying etiology in almost all age-related degenerative diseases (Franceschi et al., 2000). All joint structures including articular cartilage, synovial membrane, subchondral bone, and ligaments become involved in orchestrating the inflammation. Activated MMPs lead to progressive catabolic events in OA (Shen et al., 2017) which probably also hinder the effective repair by the stem or progenitor cells reported to be present within the joint tissue including infrapatellar fat stem cells (Stocco et al., 2019) and synovial ligament stem cells (Amemiya et al., 2019).

Mesenchymal stem cells (MSCs) derived from various sources and/or their secretory factors are used in the management of many chronic diseases including OA. The hWJSCs derived from the umbilical cord are reported to have several advantages such as hypoimmunogenicity, anti-inflammatory, and wide differentiation potential compared to other existing MSCs (Fong et al., 2010; Gauthaman et al., 2012). This property may be an advantage to overcome immune rejection upon transplantation, reduce local inflammation and contribute to the overall tissue recovery. However, controlling the underlying inflammatory activity in OA is essential for the stem cells to present within the joint structures or from other sources to integrate and contribute to cartilage repair or regeneration.

*Nigella sativa* (NS) is a medicinal plant and TQ, its main active chemical component is reported to have analgesic, diuretic, antihypertensive, antidiabetic, anticancer, immunomodulatory, anti-inflammatory and antioxidant properties (Shuid et al., 2012). Given the role of inflammation in age-related degenerative diseases and the anti-inflammatory properties of both naïve MSCs as well as the phytochemical TQ, we in the present study analyzed the effect of TQ on MSCs derived from OA patients using *in vitro* and *in silico* studies. Additionally, we evaluated the role of TQ and inflammation in age-related degenerative diseases using IPA and identified the precise molecular targets of TQ using SwissTargetPrediction.

## Materials and Methods

### Isolation and Culture of Human BM-MSCs

Bone marrow aspirates (∼5–6 ml) were collected from OA patients (*n* = 10) who underwent total knee replacement at the Department of Orthopaedics, King Abdulaziz University Hospital, Jeddah, following informed consent. The samples collected in heparinized tubes were transferred to the lab and processed immediately under sterile conditions according to our earlier established protocols (Gari et al., 2016; Kalamegam et al., 2016). Briefly, the bone marrow aspirate (2 ml/T-175 cm^2^ tissue culture flask) was cultured using Dulbecco’s Modified Eagle’s medium (DMEM; Life Technologies, Thermo Fisher Scientific, Waltham, MA, United States), supplemented with 10% fetal bovine serum (FBS; Life Technologies, Thermo Fisher Scientific, Waltham, MA, United States), 2 mM GlutaMax and antibiotics (penicillin (50 IU), streptomycin (50 μg/ml). The freshly plated bone marrow aspirate in the complete growth medium was cultured in a 5% carbon dioxide (CO2) incubator with humidified atmospheric air at 37°C.

### Flow Cytometry Analysis of CD Markers

BM-MSCs related CD markers expression was analyzed from cells of early passages (P1-P3) using FACS analysis as reported earlier (Kalamegam et al., 2016). Briefly, the cells were trypsinized upon reaching 70% confluence, using 0.25% Trypsin-EDTA (Life Technologies, Thermo Fisher Scientific, Waltham, MA, United States), centrifuged at 500 *g* × 5 min. The cell pellet was reconstituted in 3% FBS and aliquots containing 1 × 10^5^ cells/tube were used to screen for MSC related CD markers. The antibodies used were as follows: MSC isotype cocktail (negative control); MSC cocktail 1 (containing CD45-APC, CD105-FITC, and CD73-PERCP) and MSC cocktail 2 (containing CD29-PERCP, CD34-PE, CD44-PECy7, and CD90-FITC). Respective CD markers cocktail was added to the individual samples and incubated in the dark at 4°C for 30 min.

### Cell Morphology

The morphology of BM-MSCs isolated from the OA patients was analyzed to understand the biological characteristics and the effect of TQ. Briefly, 2 × 10^4^ cells/well were plated in a 24-well plate and allowed to attach overnight. Fresh culture medium was added to the cells the next day and the culture continued using the standard culture conditions for 24, 48, and 72 h. The effect of TQ on the morphology of BM-MSCs derived from OA patients was evaluated by treating the cells plated as above and using different concentrations of TQ (100 nM, 300 nM, 1 μM, 3 μM, and 5 μM) for 24, 48, and 72 h. Phase-contrast images were obtained from each experimental arms at respective time points using inverted phase-contrast optics (Nikon, Tokyo, Japan).

### Cell Metabolic Activity

#### MTT Assay

The proliferation of BM-MSCs isolated from OA patients and the effect of TQ was evaluated based on the cell metabolic activity. Briefly, 2 × 10^4^ cells/well were plated in a 24-well plate and allowed to attach overnight. Fresh culture medium was added to the cells the next day and culture continued using standard culture conditions with TQ at different concentrations (100 nM, 300 nM, 1 μM, 3 μM, and 5 μM) for 24, 48, and 72 h. At the end of each time point, the spent medium was removed and replaced with 200 μl of fresh medium containing 20 μl of MTT reagent (3-(4,5-dimethylthiazol-2-yl)-2,5-diphenyl tetrazolium bromide). The cells were incubated under the standard culture conditions for 4 h and the mitochondrial hydrogenases present within the metabolically active cells helped in the reduction of the MTT reagent. The medium was then removed and the intracellular formazan crystals, that formed due to the reduction of MTT reagent, were solubilized using dimethyl sulfoxide (DMSO, 200 μl). The plate was maintained in the dark for 30 min and then the absorbance at 570 nm with a reference wavelength at 630 nm was measured using a spectrophotometer (SpectraMax i3 Multimode reader, Molecular Devices, United States). The percentage of inhibition was calculated as control/Test-control^∗^100 and IC50 was estimated as reported earlier (Khan et al., 2016).

#### CellTiter-Blue^®^ Assay

Briefly, 1 × 10^4^ cells/well were plated in a 96-well plate and allowed to attach overnight. Fresh culture medium containing TQ (100 nM – 5 μM) was added to the cells the next day and the cells cultured for 24, 48, and 72 h as above. At the end of each time point, 20 μl CellTiter^®^-Blue cell viability reagent was added to each well and incubated for further l 2 h to allow the reduction of resazurin to highly fluorescent resorufin. The fluorescence was measured at 590 nm using the SpectraMax^®^ i3x Multi-Mode microplate reader (Molecular Devices, LLC, San Jose, CA, United States). The values were plotted against drug concentrations and IC50 was determined (Ibrahim et al., 2019).

### Differentiation Potential of BM-MSCs

#### Adipocytic Differentiation

The BM-MSCs (5 × 10^4^ cells/well) were seeded into a 6-well plate and cultured to obtain 60% confluence in a complete culture medium. The cells were then differentiated using StemPro^®^ adipocyte differentiation kit (A10070-01, Thermo Fisher Scientific). The cells for adipocytic differentiation were cultured in basal medium fortified with adipocytic supplement (StemPro^®^) for up to 14 days with fresh media change every 72 h. The control cells were cultured using the differentiation basal medium alone. Following differentiation, the cells were fixed in 4% formaldehyde solution for 30 min, rinsed with PBS twice and stained with Oil Red O (Sigma) to visualize the lipid vacuoles.

#### Chondrocytic Differentiation

The BM-MSCs (5 × 10^4^ cells/well) were seeded into a 6-well plate and differentiated along the chondrocytic lineage using StemPro^®^ chondrocyte differentiation kit (A10071-01, Thermo Fisher Scientific). The basal differentiation medium was supplemented with chondrogenic supplement (StemPro^®^) and the cells were cultured for up to 21 days with fresh media change every 72 h. The differentiated cells were then fixed in 4% formaldehyde solution for 30 min and stained with freshly prepared toluidine blue solution and analyzed for positive staining using light microscopy.

#### Osteoblastic Differentiation

The BM-MSCs (5 × 10^4^ cells/well) were seeded into a 6-well plate and differentiated along the osteoblastic lineage using StemPro^®^ osteoblast differentiation kit (A10072-01, Thermo Fisher Scientific). The basal differentiation medium was fortified with osteogenic supplement (StemPro^®^) and the cells were cultured for up to 21 days with fresh medium every 72 h. The differentiated cells were then fixed in 4% formaldehyde solution for 30 min, stained with Alizarin red (Sigma) solution, and analyzed for positive staining using light microscopy.

### Gene Expression Assay

The effect of TQ on inflammation and apoptosis-related genes in BM-MSCs isolated from the OA patients was evaluated using quantitative real-time polymerase chain reaction (qRT-PCR). Briefly, 3 × 10^5^ cells/T25 cm^2^ flask was plated and treated with two optimal concentrations of TQ (1 and 3 μM) for 48 h. The total RNA was isolated after 48 h using a Qiagen RNeasy kit (Qiagen, Germany) according to the manufacturer’s instructions. First-strand cDNA was synthesized with random hexamers using reverse transcriptase kit (Promega, Madison, WI, United States). qRT-PCR was performed using SYBRGreen master mix (Life Technologies, Thermo Fisher Scientific, Waltham, MA, United States). The following genes related to inflammation (*IFN-*γ, *TNF-*α, *COX-2*, *IL-6*, *IL-8*, *IL-16*, *IL-12A*, *IL-4*, and *IL-10*) and apoptosis (*BAX*, *BCL2*, and *SURVIVIN*) were analyzed using StepOnePlus^TM^ real-time PCR system (Thermo Fisher Scientific, Waltham, MA, United States). Primers were obtained from earlier published reports and the primer sequences are shown in Table 1.

**TABLE 1:** The genes and primer sequences used for real-time quantitative reverse transcription PCR.

Genes	Primer sequence
*GAPDH*	F: 5′GCACCGTCAAGGCTGAGAAC 3′ R: 5′ GGATCTCGCTCCTGGAAGATG 3′
*IFN-*γ	F: 5′-CCCTCACACTCAGATCATCTTCT-3′ R: 5′-GCGTTGGACATTCAAGTCAG-3′
*TNF-*α	F: 5′ GGTGCTTGTTCCTCAGCCTC 3′ R: 5′ CAGGCAGAAGAGCGTGGTG 3′
*COX-2*	F: 5′TTCAAATGAGATTGTGGGAAAATTGCT 3′ R: 5′ AGATCATCTCTGCCTGAGTATCCTT 3′
*IL-6*	F: 5′ CCACTCACCTCTTCAGAA 3′ R: 5′GCGCAAAATGAGATGAGT 3′
*IL-8*	F: 5′-AGACAGCAGAGCACACAAGC-3′ R: 5′-ATGGTTCCTTCCGGTGGT-3′
*IL-16*	F: 5′-TAGTCCTTCCTACCCAATTTCC-3′ R: 5′-TTGGTCCTTAGCCACTCCTTC-3′
*IL-12A*	F: 5′-CACTCCCAAAACCTGCTGAG-3′ R: 5′-TCTCTTCAGAAGTGCAAGGGTA-3′
*IL-4*	F: 5′-TGGATCTGGGAGCATCAAGGT-3′ R: 5′-TGGAAGTGCGGATGTAGTCAG-3′
*IL-10*	F: 5′-GCTCTTACTGACTGGCATGAG-3′ R: 5′-CGCAGCTCTAGGAGCATGTG-3′
*BAX*	F: 5′GGCTGGGATGCCTTTGTG 3′ R: 5′ CAGCCAGGAGAAATCAAACAGA 3′
*BCL-2*	F: 5′ TGGAGCTGCAGAGGATGATTG 3′ R: 5′ GCTGCCACTCGGAAAAAGAC 3′
*SURVIVIN*	F: 5′-ACCAGGTGAGAAGTGAGGGA-3′ R: 5′-AACAGTAGAGGAGCCAGGGA-3′

*F, forward primer; R, reverse primer.*

### SwissTargetPrediction

To identify the beneficial effects of TQ in relation to AD and OA, their targets prediction was performed using the SwissTargetPrediction web tool^1^ with an update on bioactivity data, retrained and redefined similarity thresholds (Daina et al., 2019). The ligand-based target prediction was performed based on the similarity between the query molecule and the compiled curated collection using 2D and 3D similarity measures within a larger bioactivity data of ChEMBL version 23. A combined score of higher than 0.5 indicates that the molecules share a common protein target. In reverse screening, the combined score helps to calculate the probability to target a given protein. The dual based reverse screening demonstrates high performance in predicting macromolecular targets.

### Ingenuity Pathway Analysis

Functional analysis was used to predict the molecular interactions of TQ with OA, and age-related degenerative diseases using IPA software (Qiagen, United States). The endogenous chemical TQ was imported into the IPA software to generate the interacting molecules. The molecules were then used in the expression analysis to predict signaling mechanisms, targets and their association to TQ, OA, and age-related degenerative diseases using direct or indirect relationship. Network predictions based on the input of molecules were generated using algorithms contained in the Ingenuity Knowledge Base. Fischer’s Exact test was carried out to calculate the *p*-value, indicating the probability of each biological function associated with the network.

### Statistical Analysis

Statistical analyses were performed using the statistical package for social sciences (SPSS) version 21. Students’ *t*-test or One-way ANOVA was used for analysis between controls and treated groups. The values were expressed as mean ± SEM (Standard error of the mean) from a minimum of three experimental replicates. Asterisk (^∗^) indicates the statistical significance of *P* < 0.05.

## Results

### CD Marker Characterization of Derived BM-MSCs

**FIGURE 1:**
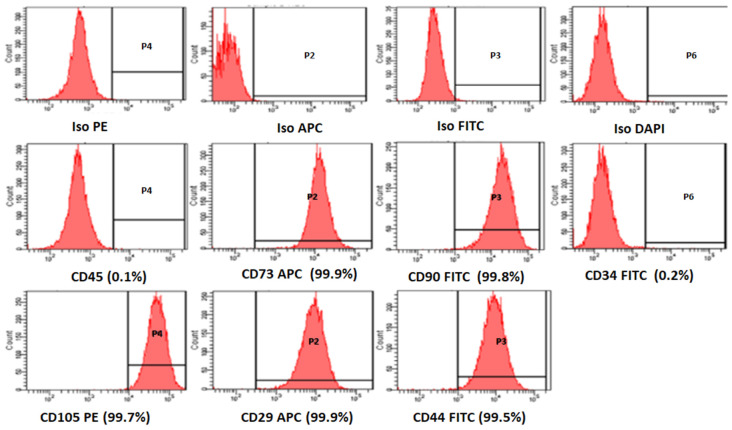
Representative histogram of the CD surface markers on BM-MSCs derived from osteoarthritis patients, using fluorescent activated cell sorting (FACS). The MSC positive surface CD markers namely CD73, CD90, CD105, CD29, CD44, and the MSC negative surface CD markers namely CD34, CD45 are shown. The independent CD marker antigens were tagged with different fluorochromes. All the MSC positive CD surface markers demonstrated more than 90% positivity, and their histograms were considerably shifted to the right compared to their respective isotype controls. ISO, isotype; PE, phycoerythrin; APC, allophycocyanin; FITC, Fluorescein isothiocyanate; DAP-4′,6-diamidino-2-phenylindole.

The BM-MSCs were successfully isolated with all samples obtained from OA patients. These BM-MSCs demonstrated a high expression of positive MSCs related CD surface markers namely CD29 (99.9%), CD44 (99.5%), CD73 (99.9%), CD90 (99.8%), and CD105 (99.7%) in early passages (Figure 1). These cells were negative for the hematopoietic markers namely CD34 (0.2%) and CD45 (0.1%) (Figure 1).

### BM-MSCs Morphology and Growth Characteristics

**FIGURE 2:**
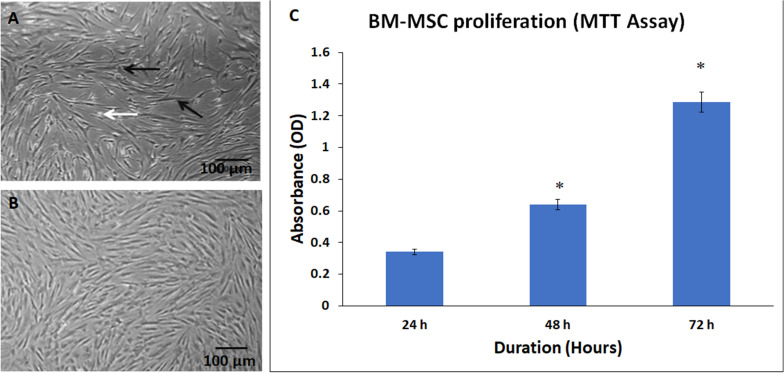
Phase contrast images showing primary cultures of bone marrow mesenchymal stem cells (BM-MSCs) derived from osteoarthritis patients at **(A)** early passage (P0) and **(B)** later passage (P5). Black and white arrows indicate the adherent fibroblast cell and dead floating cell, respectively. **(C)** Normal cell proliferation of BM-MSCs at 24, 48, and 72 h. The values are expressed as mean ± SEM of three different experiments. * indicates statistical significance (*p* < 0.05).

The stem cell precursors/nucleated cells in marrow aspirates adhered and formed small colonies of cells by days 5–7 (colony forming units). These cells rapidly expanded and reached 65–70% confluence by day 10. At initial passages, these cells appeared as thin spindle-shaped cells resembling fibroblasts (Figures 2A,B). The non-adherent cells, dead cells, and RBCs were removed with medium changes thus allowing expansion of the BM-MSCs.

The derived BM-MSCs showed good expansion of cells with subsequent passages. There was a time-dependent increase in cell numbers at 24, 48, and 72 h, as demonstrated by the MTT assay (Figure 2C). The increase in the percentage of cell proliferation was 87.39% and 277.42% at 48 and 72 h compared to 24 h and these increases were statistically significant (Figure 2C).

### Differentiation Potential of BM-MSCs

**FIGURE 3:**
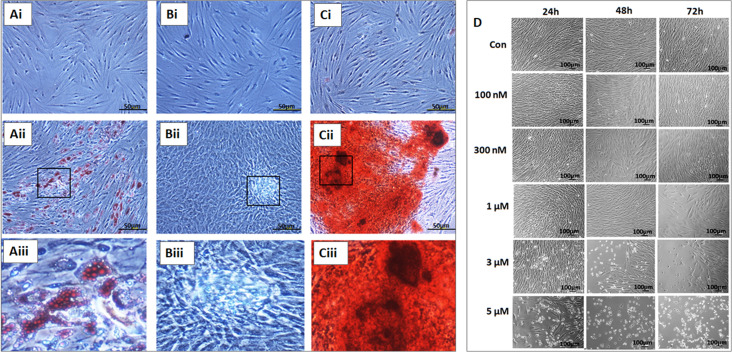
*In vitro* differentiation images of the bone marrow mesenchymal stem cells (BM-MSCs) into adipocytes **(Ai–Aiii)**, chondroblasts **(Bi–Biii)** and osteobalsts **(Ci–Ciii)** respectively. **(Ai,Bi,Ci)** represent respective controls; **(Aii,Bii,Cii)** are the differentiated images; **(Aiii,Biii,Ciii)** are the magnified images of the boxed area. **(D)** Phase contrast images of the BM-MSCs derived from osteoarthritis patients treated with different concentrations (100 nM, 300 nM, 1, 3, and 5 μM) of thymoquinone (TQ) for 24h, 48h, and 72h. An increase in cell death of BM-MSCs with time following treatment with higher concentrations of TQ was noted.

The BM-MSCs showed differentiation into adipocytes, osteoblasts, and chondrocytes following culture in the respective differentiation medium. The cells cultured using StemPro^®^ adipocyte differentiation kit (A10070-01 Thermo Fisher Scientific) demonstrated lipid vacuolations as early as 10 days and their numbers and size increased when cultured up to 21 days. These differentiated cells demonstrated positive staining with oil red O (Figures 3Ai–iii). The BM-MSCs cultured using StemPro^®^ chondrocyte differentiation kit (A10071-01, Thermo Fisher Scientific) demonstrated loss of fibroblastic morphology and attained rounded or polygonal shape. When cultured for 21 days, they formed small cell clusters which demonstrated positive staining with Alcian blue compared to the control (Figures 3Bi–iii). The BM-MSCs cultured using StemPro^®^ chondrocyte differentiation kit (A10072-01, Thermo Fisher Scientific) demonstrated granular cell deposits as early as day 14 and they became denser at 21 days of culture. These differentiated cells demonstrated positive staining with Alizarin red, indicative of calcium mineralization (Figures 3Ci–iii).

### Effect of Thymoquinone on BM-MSCs Morphology

Phase-contrast microscopy of BM-MSCs treated with TQ at different concentrations ranging from 100 nM to 5 μM for 48 h demonstrated various morphological changes including loss of their characteristic fibroblastic shape, cell shrinkage and membrane damage resulting in death. Mild to moderate increase in cell numbers were observed at lower concentrations while higher concentrations caused more cell death and a decrease in cell numbers (Figure 3D).

### Effect of Thymoquinone on BM-MSCs Metabolic Activity

**FIGURE 4:**
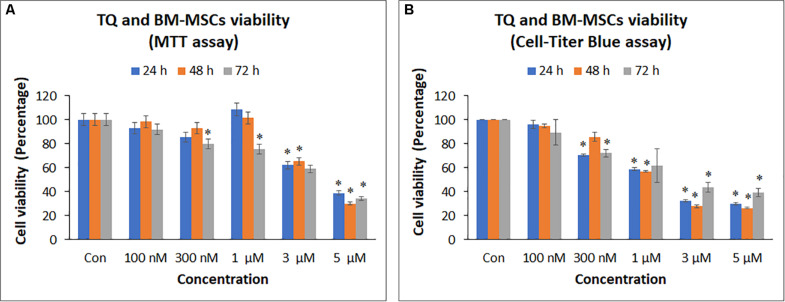
Cell proliferation [**(A)** MTT assay; **(B)** Cell-titer Blue assay] of bone marrow mesenchymal stem cells (BM-MSCs) derived from osteoarthritis patients treated with different concentrations (100 nM, 300 nM, 1, 3, and 5 μM) of thymoquinone (TQ) for 24h, 48h, and 72h. Mean decreases in cell proliferation were observed in increasing concentrations of TQ. Some of these decreases with higher concentrations were statistically significant compared to the control. The values are expressed as mean ± SEM of three different experiments. *indicates statistical significance (*p* < 0.05).

3-(4,5-dimethylthiazol-2-yl)-2,5-diphenyl tetrazolium bromide assay of BM-MSCs treated with TQ at different concentrations (100 nM, 300 nM, 1 μM, 3 μM, and 5 μM) for 24, 48, and 72 h showed a decrease in cell viability with increasing concentrations of TQ compared to the control (Figure 4A). However, only the decreases observed in cell viability with 3 μM (37.84%) and 5 μM (61.08%) of TQ at 24 h; 3 μM (34.72%) and 5 μM (69.76%) of TQ at 48 h; and 300 nM (20.04%), 1 μM (24.40%), 3 μM (40.91%), and 5 μM (65.67%) at 72 h of TQ were statistically significant (*P* < 0.05) compared to their respective controls (Figure 4A). The IC50 value at 48 h following MTT assay was 3.78 ± 0.52 μM.

CellTiter Blue^®^ assay of BM-MSCs treated with TQ at different concentrations (100 nM – 5 μM) also showed a decrease in cell viability with increasing concentrations of TQ compared to the control (Figure 4B). The decrease in cell viability observed with 300 nM (29.49%), 1 μM (41.05%), 3 μM (67.71%), and 5 μM (70.20%) of TQ at 24 h; 1 μM (42.97%), 3 μM (72.22%), and 5 μM (73.67%) of TQ at 48 h; and 300 nM (27.80%), 1 μM (37.94%), 3 μM (56.40%), and 5 μM (60.47%) of TQ at 72 h were statistically significant (*P* < 0.05) compared to their respective controls (Figure 4B). The IC50 value at 48 h following CellTiter Blue^®^ assay was 1.74 ± 0.34 μM.

### Gene Expression Analysis

**FIGURE 5:**
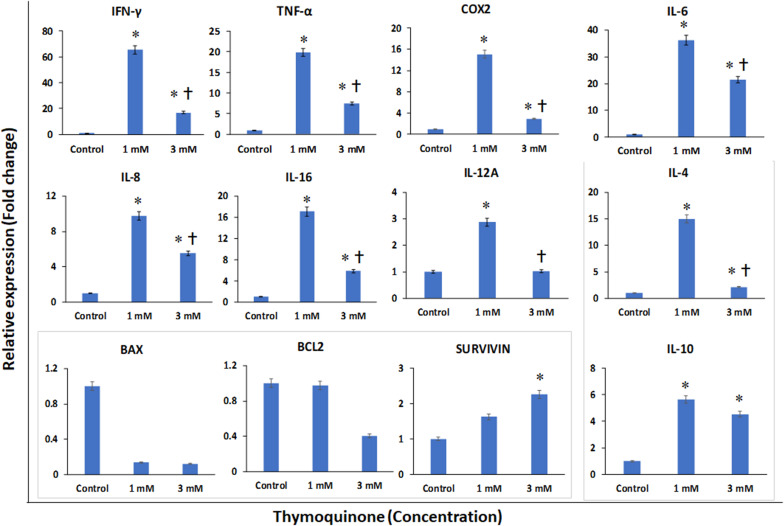
Gene expression analysis of pro-inflammatory (INF-γ, TNF-α, COX-2, IL-6, IL-8, IL-16, and IL-12A); anti-inflammatory (IL-4 and IL-10) and apoptotic (BAX, BCL2 and SURVIVIN) related genes in bone marrow mesenchymal stem cells (BM-MSCs) derived from osteoarthritis patients treated with 1 μM and 3 μM of thymoquinone (TQ) for 48h, using quantitative real-time PCR. Data analysis and relative quantitation were performed using the comparative Ct method (ΔΔCt). ^∗^ and † indicates statistical significance (*p* < 0.05) compared to the control and TQ 1 μM respectively.

The BM-MSCs treated with TQ (1 and 3 μM) for 48 h were evaluated for the expression of inflammation and apoptosis-related genes. The following pro-inflammation related genes namely *INF-*γ, *TNF-α, COX-2*, *IL-6*, *IL-8*, *IL-16*, and *IL-12A* showed upregulation compared to the control (GAPDH). The fold increases in gene expression were as follows: *INF-*γ by 65.46 and 16.89; *TNF-α* by 19.80 and 7.41; *COX-2* by 15.05 and 2.94; *IL-6* by 36.20 and 21.50; *IL-8* by 9.72 and 5.51; *IL-16* by 17.08 and 5.86; and *IL-12A* by 2.87 and 1.02 following treatment with TQ 1 and 3 μM, respectively (Figure 5). However, the expression of these pro-inflammatory genes was more upregulated at a lower concentration (1 μM) than at 3 μM of TQ (Figure 5). The anti-inflammatory genes namely *IL-4* and *IL-10* were upregulated compared to the control. The fold increases for *IL-4* were 14.95 and 20.12; and for *IL-10* the fold increases were 4.53 and 5.65, following treatment with TQ 1 and 3 μM, respectively (Figure 5). The pro-apoptotic *BAX* gene demonstrated an insignificant (low) expression compared to the control. In contrast, both the anti-apoptotic *BCL2* and *SURVIVIN* genes demonstrated an upregulation compared to the control. The fold increases for *BCL2* were 12.97 and 14.41, and for *SURVIVIN* the fold increases were 10.63 and 22.26 following treatment with TQ 1 and 3 μM, respectively (Figure 5).

### SwissTargetPrediction

**FIGURE 6:**
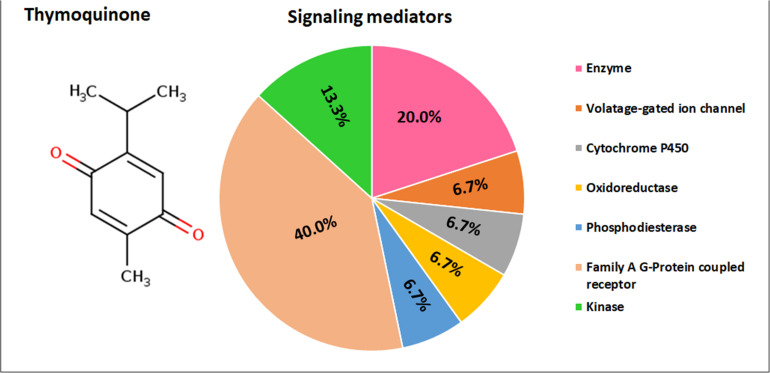
SwissTargetPrediction showing the top biomolecular targets for thymoquinone.

Thymoquinone is identified to target the following molecular/biochemical pathways namely, G-protein coupled receptor; transcription factors; oxidoreductase; lyase; cytochrome P450; kinase; ligand-gated ion channels and hydrolase. Although more bioactive targets were curated based on 2D and 3D similarity measures, the above targets are among the top 15 curated based on our query molecule (Figure 6). Most of the protein targets identified using the latest SwissTargetPrediction (2019 version) have high probability value (Figure 6) and are potential therapeutic targets for age-related degenerative diseases including OA.

### Ingenuity Pathways Analysis of Inflammation Network

**FIGURE 7:**
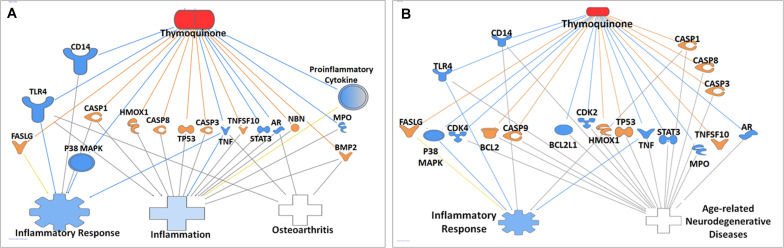
Ingenuity pathway analysis of genes/molecules that are regulated by thymoquinone in relation to Alzheimer’s disease and inflammation **(A)**, and osteoarthritis and inflammation **(B)**.

Ingenuity pathway analysis core analysis of TQ in relation to inflammation and its association with OA (Figure 7A) and age-related degenerative diseases (Figure 7B) using Ingenuity Knowledge Base reference sets identified top canonical pathways, diseases, upstream regulators, biological and toxicological functions. IPA of TQ and OA identified TGF-β signaling, receptor-activated of NF-kB (RANK) signaling and IL signaling. Diseases of the joints associated with inflammation included OA, rheumatoid arthritis (RA), rheumatic disease and connective tissue tumor. Screening of OA or aging and their closely related diseases led to the identification of 36 molecules that are involved in apoptosis, cell cycle regulation, cell signaling, cytokines, chemokines, and growth factors (Table 2). Similarly, IPA of TQ and aging, in particular neurodegeneration, identified few canonical pathways directly related to synaptogenesis signaling, synaptic long term depression, synaptic long term potentiation, neuroinflammation signaling and neuroprotective role of Thimet oligopeptidase (THOP1). The neurological conditions associated with inflammation included AD, dementia, basal ganglia disorders, progressive neurological diseases, amyotrophic lateral sclerosis, neuromuscular disease, motor neuropathy, glioma, glioblastoma, astrocytoma, and neuroblastoma (Table 2).

**TABLE 2:** The common interacting molecules in Alzheimer’s disease, osteoarthritis, and closely related diseases.

	Diseases	Interacting molecules
Age-related degenerative diseases associated with inflammation	Alzheimer’s Disease (25 molecules) Dementia (26 molecules) Basal Ganglia Disorders (18 molecules) Progressive neurological diseases (18 molecules)	**Apoptosis regulators:** (BAX, BCL2, BCL2L1, CASP1, CASP3, CASP8, CASP9, TP53, and XIAP) **Cell cycle regulators:** (CD14, CDK2, CDK4, and CDKN1A) **Cytokines, chemokines, growth factors:** (CXCR4, IL6, IL10, TNF, and VEGF) **Cell signaling:** (NFkB, STAT3, JNK, Akt, and MAPK1) **Others:** (AR, COL1A1, COL3A1, E2F1, HDL-cholesterol, HMOX1, MPO, PKM, TLR4)
	Osteoarthritis (10 molecules) Rheumatoid arthritis (23 molecules) Joint inflammation (27 molecules) Connective tissue disorders (21 molecules)	**Apoptosis regulators:** (AX, BCL2, BCL2L1, BIK, BIRC5, CASP1, CASP3, CASP8, CASP9, FASLG, TNFSF10, TP53, and XIAP) **Cell cycle regulators:** (CDKN1A) **Cytokines, chemokines, growth factors:** (CXCR4, IL6, IL10, TNF, and VEGF) **Cell signaling:** (Akt, ERK, JNK, and STAT3) **Others:** (AR, BMP2, COL3A1, E2F1, HMOX1, LDL-cholesterol, MPO, NBN, and TLR4)

## Discussion

Chronic inflammation is one of the common underlying pathology leading to articular cartilage tissue damage in OA. The poor cartilage regeneration capacity in OA and the associated inflammation recruits endogenous molecules and activates a cascade of pro-inflammatory cytokines, which in turn cause cartilage degradation, joint structure deformity, and function (Scanzello, 2017). Although the BM-MSCs were derived from OA patients, these cells demonstrated the biological properties of having fibroblastic phenotype, adherence to tissue culture plastic and differentiation into adipocytes, osteoblasts, and chondrocytes, thus fulfilling the stipulated minimal criteria of MSCs (Dominici et al., 2006). The BM-MSCs expansion in culture either in the presence or absence of TQ was indirectly assessed based on the cell metabolic activity (MTT and CellTiter-Blue^®^ assays). It was evident that TQ at higher concentrations (5 μM) led to morphological changes and cell death compared to lower concentrations. In the present study, we identified that the IC50 concentration of TQ to be 3 μM based on the mean concentrations from two different assays (MTT and CellTiter-Blue^®^) at 48 h. The concentrations of TQ and their cellular effects varied from 0.1 to 100 μM based on the purity of the compound, the cell types used and the nature of the studies (Muralidharan-Chari et al., 2016; Aziz et al., 2018; Bordoni et al., 2019).

In the present study, we observed that the pro-inflammatory genes namely *IFN-*γ, *TNF-*α, *COX-2*, *IL-6*, *IL-8*, *IL-16*, and *IL-12A* were upregulated compared to the control. However, compared to the lower concentration (1 μM) of TQ, these pro-inflammatory genes were downregulated at the higher concentration (3 μM). TQ was reported to downregulate pro-inflammatory genes such as *IL-1*, *TNF-*α, and toll-like receptors (*TLR2*, *TLR4*) and attenuate rheumatoid arthritis *via* the NFkB pathway (Arjumand et al., 2019). Earlier *in vitro* and *in vivo* animal studies have reported that TQ reduces pro-inflammatory cytokines such as *TNF-*α, *COX-2*, and *IL-6* in the brain, Schwann cell culture and peritoneal mast cells (Chen et al., 2016; Ikhsan et al., 2018). TQ is also reported to offer neuroprotection by reducing the pro-inflammatory cytokines in *IFN-*γ activated microglial cells *via* NF-kB dependent signaling (Cobourne-Duval et al., 2018). Interestingly, we also observed that the anti-inflammatory cytokines *IL-4* and *IL-10* were upregulated compared to the control at both 1 and 3 μM. As such TQ may impart the dual benefit of upregulating the anti-inflammatory cytokines and partially down-regulating the pro-inflammatory cytokines. In addition to anti-inflammatory effects, TQ was also reported to have anti-cancer and antioxidant effects. For example, in combination with cisplatin, TQ demonstrated an increased expression of the pro-apoptotic BAX and decreased anti-apoptotic *BCL2* in a ovarian carcinoma (SKOV3) cell line (Liu et al., 2017). In the present study we observed that *BAX* gene expression was much decreased compared to BCL2 with 3 μM concentration of TQ. Furthermore, *SURVIVIN* gene expression was upregulated with both 1 and 3 μM concentrations of TQ indicating that TQ, in fact, might offer protection to normal cells, unlike the karyotypically abnormal cancer cells. However, at a further higher concentration of TQ (5 μM) we observed more cell death, and hence it will be prudent to study the effectiveness of TQ on other types of stem cells and normal tissue-specific cells to determine the optimal concentration before its therapeutic consideration.

*In silico* analysis using Ingenuity Knowledge Base identified decreased MPO enzyme and increased heme oxygenase 1 (HMOX1) in both OA and aging. MPO enzyme released following neutrophil oxidative burst response is reported to cause non-specific tissue damage in AD (Gellhaar et al., 2017). Interestingly, a 7,8-Dihydroflavaone commonly present in plants protects against OA by increasing HMOX1 (Cai et al., 2019) and also increased spatial memory in a rodent model of aging (Castello et al., 2014).

Further, we also noted TQ interaction with caspases, MMPs, and ROS in both OA and age-related neurodegenerative diseases. Activation of caspases, MMPs, and oxidative stress are associated with cell damage including those of chondrocytes in OA and neuronal cells in age-related neurodegenerative diseases (Youssef et al., 2018; Li et al., 2019). The link between oxidative stress and inflammation is well established and also the fact that inflammation is one of the pathological states in both OA and age-related neurodegenerative diseases. In response to oxidative stress, the inflammatory cells are reported to release NF-κB mediated pro-inflammatory mediators. More evidence points to the association of ROS and neuroinflammation leading to neurodegeneration (Rojo et al., 2014). TQ is also reported to reduce oxidative stress, mediate MAPK and apoptosis pathways and protect against neurotoxicity in rats *via* its anti-oxidant and anti-apoptotic effects (Tabeshpour et al., 2019). As such targeting the mediators of inflammation will, therefore, be an effective strategy in the management of age-related degenerative diseases.

Cyclin D4 activity is important for the G1/S transition of the cell cycle; however, their levels were reported to be disproportionately higher in age-related degenerative diseases. Synthetic CDK4 inhibitors demonstrated the protection of primary rat cortical neurons and PC12 cells that were deprived of nerve growth factor or exposed to β-amyloid toxicity (Sanphui et al., 2013). Inflammatory agents like LPS interact with toll-like receptor 4 (TLR4) and activate MAPK and its related family including extracellular regulated kinase, c-JNK, and p38MAPK (Xu et al., 2017). Interestingly, TQ is known to reduce the ROS levels by reduction of NADPH-oxidase, and also exerts anti-inflammatory effects by activation of adenosine-monophosphate activated protein kinase and Sirtuin-1 in activated BV2 microglial cells (Velagapudi et al., 2017). TQ also inhibits the inflammatory effects of the proinflammatory cytokine TNF-α by activation of p38/JNK via apoptosis-regulated signaling kinase 1 in rheumatoid arthritis synovial fibroblasts (Umar et al., 2015). Also, a recent study reported that TQ exerts anti-inflammatory effects by reduction of TNF-α, TLR-2, TLR-4, and Interleukin-1 in rheumatoid arthritis (Arjumand et al., 2019). Furthermore, the IL-1β induced inflammation in chondrocytes in OA is inhibited by TQ *via* suppression of NF-kB and MAPK signaling pathways (Wang et al., 2015).

Apart from the standard use of cyclooxygenase (COX) inhibitors or cholinesterase inhibitors for OA and age-related neurodegenerative disease (AD), respectively, alternative therapies including nutritional supplements and herbal preparations are also commonly used to reduce inflammation and improve general health (Tian et al., 2010; Wang, 2013). TQ, the active ingredient of *Nigella sativa* is reported to have anti-inflammatory and antioxidant properties which therefore can complement conventional therapies for additive effects (Jakaria et al., 2018). We also observed in the present study that TQ at lower concentrations did not affect the proliferation of BM-MSCs derived from OA patients. The molecular targets of TQ as identified by SwissTargetPrediction include mainly the enzymes (kinase, ligase, oxidoreductase, and phosphatase) and family of G-protein coupled receptors. Further studies on the effect of TQ or the paracrine molecules of MSCs on the above molecular targets will help to understand the precise mechanism of TQ and/or MSCs secretions in limiting the pathology in age-related degenerative diseases.

The secretions from MSCs are also reported to have immunomodulatory, anti-inflammatory, anti-apoptotic, neuroprotective and neurotrophic effects (Vizoso et al., 2017). hWJSCs isolated from the human umbilical cord have both embryonic and MSC properties in addition to several advantages compared to other existing MSCs. The hWJSCs have also been differentiated into many different cell types including osteocytes, chondrocytes, and neurons (Fong et al., 2010, 2012). Besides, the hWJSCs have immunomodulatory, anti-inflammatory, anti-apoptotic, anti-cancer effects (Kalamegam et al., 2018b). Therefore, in addition to conventional pharmacological agents, the use of stem cells/stem cell factors and/or TQ as therapeutics will help regeneration of the damaged tissue and also reduce the pathological changes associated with age-related degenerative diseases.

## Conclusion

Aging is inevitable and inflammation is a common pathology in most of the age-related degenerative diseases. The present study revealed that TQ has moderate anti-inflammatory effects and therefore has the potential to be used in therapeutics. TQ, either alone or in combination with conventional pharmacological agents will help in reducing the inflammation associated with inflammatory joints and age-related degenerative diseases. However, confirmation of our *in vitro* and *in silico* results with *in vivo* animal studies will help to understand the underlying mechanism and their real therapeutic benefits.

## Data Availability Statement

The datasets generated for this study are available on request to the corresponding author.

## Ethics Statement

The studies involving human participants were reviewed and approved by Unit of BioMedical Ethics Committee of the King Abdulaziz University vide approval number 11-557. The patients/participants provided their written informed consent to participate in this study.

## Author Contributions

GK and PP were involved in conceptualization, intellectual contribution, statistical evaluation, and manuscript writing. MA is a clinician and was involved in providing clinical materials/information and intellectual support. SA, AB, FA, GK, MA-E, and PP were involved in the experimental work and data analysis. EA, MG, and MA-E were involved in co-ordination of the work, review, and editing of the manuscript. All authors contributed to the article and approved the submitted version.

## Conflict of Interest

The authors declare that the research was conducted in the absence of any commercial or financial relationships that could be construed as a potential conflict of interest.

## Footnotes

1 www.swisstargetprediction.ch

## Abbreviations

AD: Alzheimer’s Disease
ANOVA: analysis of variance
APC: allophycocyanin
BAX: BCL2 associated X
BCL2: B-Cell Lymphoma 2
BM-MSCs: bone-marrow mesenchymal stem cells
CD: cluster of differentiation
ChEMBL: Chemical database of European Molecular Biology Laboratory
c-JNK: c-JunN-terminal kinases
COX-2: cyclooxygenase-2
FACS: fluorescent activated cell sorting
FITC: fluorescein isothiocyanate
HMOX1: heme oxygenase 1
hWJSCs: human Wharton’s Jelly stem cells
IC50: half maximal inhibitory concentration
IFN- γ: interferon-gamma
IL: interleukin
IPA: ingenuity pathway analysis
MAPK: mitogen-activated protein kinase
MMPs: matrix metalloproteinases
MPO: myeloperoxidase
MTT: 3-(4,5-dimethylthiazol-2-yl)-2,5-diphenyl tetrazolium bromide
NADPH: nicotinamide adenine dinucleotide phosphate
NF- κ B: nuclear factor Kappa-light-chain-enhancer of activated B cells
NS: *Nigella sativa*
OA: osteoarthritis
PE: phycoerythrin
PECy7: phycoerythrin and a cyanine dye
PERCP: peridinin-chlorophyll-protein
ROS: reactive oxygen species
SEM: standard error of the mean
TGF- β: transforming growth factor-beta
TLR: toll-like receptor
TNF- α: tumor necrosis factor-alpha
TQ: thymoquinone.

## References

[B1] Amemiya M. Tsuji K. Katagiri H. Miyatake K. Nakagawa Y. Sekiya I. (2019). Synovial fluid-derived mesenchymal cells have non-inferior chondrogenic potential and can be utilized for regenerative therapy as substitute for synovium-derived cells. *Biochem. Biophys. Res. Commun.* 523 465–472. 10.1016/j.bbrc.2019.12.068 31882120

[B2] Arjumand S. Shahzad M. Shabbir A. Yousaf M. Z. (2019). Thymoquinone attenuates rheumatoid arthritis by downregulating TLR2, TLR4, TNF-α, IL-1, and NFκB expression levels. *Biomed. Pharmacother.* 111 958–963. 10.1016/j.biopha.2019.01.006 30841475

[B3] Aziz N. Son Y.-J. Cho J. Y. (2018). Thymoquinone suppresses IRF-3-mediated expression of type I interferons via suppression of TBK1. *Intern. J. Mol. Sci.* 19:1355. 10.3390/ijms19051355 29751576 PMC5983753

[B4] Bordoni L. Fedeli D. Nasuti C. Maggi F. Papa F. Wabitsch M. (2019). Antioxidant and anti-inflammatory properties of Nigella sativa oil in human pre-adipocytes. *Antioxidants* 8:51. 10.3390/antiox8020051 30823525 PMC6406245

[B5] Cai D. Feng W. Liu J. Jiang L. Chen S. Yuan T. (2019). 7,8-Dihydroxyflavone activates Nrf2/HO-1 signaling pathways and protects against osteoarthritis. *Exper. Therap. Med.* 18 1677–1684. 10.3892/etm.2019.7745 31410125 PMC6676087

[B6] Castello N. A. Nguyen M. H. Tran D. J. Cheng D. Green K. N. LaFerla F. M. (2014). 7,8-Dihydroxyflavone, a small molecule TrkB agonist, improves spatial memory and increases thin spine density in a mouse model of Alzheimer disease-like neuronal loss. *PLoS One* 9:e91453. 10.1371/journal.pone.0091453 24614170 PMC3948846

[B7] Chen L. Li B. Chen B. Shao Y. Luo Q. Shi X. (2016). Thymoquinone alleviates the experimental diabetic peripheral neuropathy by modulation of inflammation. *Sci. Rep.* 6 1–11. 10.1038/srep31656 27545310 PMC4992870

[B8] Cobourne-Duval M. K. Taka E. Mendonca P. Soliman K. F. (2018). Thymoquinone increases the expression of neuroprotective proteins while decreasing the expression of pro-inflammatory cytokines and the gene expression NFκB pathway signaling targets in LPS/IFNγ-activated BV-2 microglia cells. *J. Neuroimmunol.* 320 87–97. 10.1016/j.jneuroim.2018.04.018 29759145 PMC5967628

[B9] Cole J. H. Franke K. (2017). Predicting age using neuroimaging: innovative brain ageing biomarkers. *Trends Neurosci.* 40 681–690. 10.1016/j.tins.2017.10.001 29074032

[B10] da Costa J. P. Vitorino R. Silva G. M. Vogel C. Duarte A. C. Rocha-Santos T. (2016). A synopsis on aging—theories, mechanisms and future prospects. *Ageing Res. Rev.* 29 90–112. 10.1016/j.arr.2016.06.005 27353257 PMC5991498

[B11] Daina A. Michielin O. Zoete V. (2019). Swiss target prediction: updated data and new features for efficient prediction of protein targets of small molecules. *Nucleic Acids Res.* 47 W357–W364. 10.1093/nar/gkz382 31106366 PMC6602486

[B12] Dominici M. Le Blanc K. Mueller I. Slaper-Cortenbach I. Marini F. Krause D. (2006). Minimal criteria for defining multipotent mesenchymal stromal cells. International society for cellular therapy position statement. *Cytotherapy* 8 315–317. 10.1080/14653240600855905 16923606

[B13] Fong C.-Y. Subramanian A. Biswas A. Gauthaman K. Srikanth P. Hande M. P. (2010). Derivation efficiency, cell proliferation, freeze-thaw survival, stem-cell properties and differentiation of human Wharton’s jelly stem cells. *Reprod. Biomed.* 21 391–401. 10.1016/j.rbmo.2010.04.010 20638335

[B14] Fong C.-Y. Subramanian A. Gauthaman K. Venugopal J. Biswas A. Ramakrishna S. (2012). Human umbilical cord Wharton’s jelly stem cells undergo enhanced chondrogenic differentiation when grown on nanofibrous scaffolds and in a sequential two-stage culture medium environment. *Stem Cell Rev. Rep.* 8 195–209. 10.1007/s12015-011-9289-8 21671058

[B15] Franceschi C. Bonafè M. Valensin S. Olivieri F. De Luca M. Ottaviani E. (2000). Inflamm-aging: an evolutionary perspective on immunosenescence. *Ann. N. Y. Acad. Sci.* 908 244–254. 10.1111/j.1749-6632.2000.tb06651.x 10911963

[B16] Gari M. Alsehli H. Gari A. Abbas M. Alkaff M. Abuzinadah M. (2016). Derivation and differentiation of bone marrow mesenchymal stem cells from osteoarthritis patients. *Tissue Eng. Regen. Med.* 13 732–739. 10.1007/s13770-016-0013-2 30603454 PMC6170872

[B17] Gauthaman K. Fong C.-Y. Suganya C.-A. Subramanian A. Biswas A. Choolani M. (2012). Extra-embryonic human Wharton’s jelly stem cells do not induce tumorigenesis, unlike human embryonic stem cells. *Reprod. Biomed.* 24 235–246. 10.1016/j.rbmo.2011.10.007 22196893

[B18] Gellhaar S. Sunnemark D. Eriksson H. Olson L. Galter D. (2017). Myeloperoxidase-immunoreactive cells are significantly increased in brain areas affected by neurodegeneration in Parkinson’s and Alzheimer’s disease. *Cell Tissue Res.* 369 445–454. 10.1007/s00441-017-2626-8 28466093 PMC5579172

[B19] Ibrahim S. M. Bakhashab S. Ilyas A. M. Pushparaj P. N. Karim S. Khan J. A. (2019). WYE-354 restores Adriamycin sensitivity in multidrug-resistant acute myeloid leukemia cell lines. *Oncol. Rep.* 41 3179–3188. 10.3892/or.2019.7093 30942458 PMC6489006

[B20] Ikhsan M. Hiedayati N. Maeyama K. Nurwidya F. (2018). Nigella sativa as an anti-inflammatory agent in asthma. *BMC Res. Notes* 11:8. 10.1186/s13104-018-3858-8 30340634 PMC6194640

[B21] Jakaria M. Cho D.-Y. Haque E. Karthivashan G. Kim I.-S. Ganesan P. (2018). Neuropharmacological potential and delivery prospects of thymoquinone for neurological disorders. *Oxid. Med. Cell. Long.* 2018:1209801. 10.1155/2018/1209801 29743967 PMC5883931

[B22] Kalamegam G. Abbas M. Gari M. Alsehli H. Kadam R. Alkaff M. (2016). Pelleted bone marrow derived mesenchymal stem cells are better protected from the deleterious effects of arthroscopic heat shock. *Front. Physiol.* 7:180. 10.3389/fphys.2016.00180 27252654 PMC4877393

[B23] Kalamegam G. Memic A. Budd E. Abbas M. Mobasheri A. (2018a). A comprehensive review of stem cells for cartilage regeneration in osteoarthritis. *Adv. Exper. Med. Biol.* 1089 23–36. 10.1007/5584_2018_205 29725971

[B24] Kalamegam G. Sait K. H. W. Ahmed F. Kadam R. Pushparaj P. N. Anfinan N. (2018b). Human Wharton’s Jelly stem cell (hWJSC) extracts inhibit ovarian cancer cell lines OVCAR3 and SKOV3 in vitro by inducing cell cycle arrest and apoptosis. *Front. Oncol.* 8:592. 10.3389/fonc.2018.00592 30581772 PMC6293270

[B25] Khan F. Ahmed F. Pushparaj P. N. Abuzenadah A. Kumosani T. Barbour E. (2016). Ajwa date (*Phoenix dactylifera* L.) extract inhibits human breast adenocarcinoma (mcf7) cells in vitro by inducing apoptosis and cell cycle arrest. *PLoS One* 11:158963. 10.1371/journal.pone.0158963 27441372 PMC4956039

[B26] Li D. Ni S. Miao K. S. Zhuang C. (2019). PI3K/Akt and caspase pathways mediate oxidative stress-induced chondrocyte apoptosis. *Cell Stress Chaper.* 24 195–202. 10.1007/s12192-018-0956-4 30543056 PMC6363634

[B27] Lin A. C. Seeto B. L. Bartoszko J. M. Khoury M. A. Whetstone H. Ho L. (2009). Modulating hedgehog signaling can attenuate the severity of osteoarthritis. *Nat. Med.* 15:1421. 10.1038/nm.2055 19915594

[B28] Liu X. Dong J. Cai W. Pan Y. Li R. Li B. (2017). The effect of thymoquinone on apoptosis of SK-OV-3 ovarian cancer cell by regulation of Bcl-2 and Bax. *Intern. J. Gynecol. Cancer.* 27 1596–1601. 10.1097/IGC.0000000000001064 28692636

[B29] Mobasheri A. Kalamegam G. Musumeci G. Batt M. E. (2014). Chondrocyte and mesenchymal stem cell-based therapies for cartilage repair in osteoarthritis and related orthopaedic conditions. *Maturitas* 78 188–198. 10.1016/j.maturitas.2014.04.017 24855933

[B30] Muralidharan-Chari V. Kim J. Abuawad A. Naeem M. Cui H. Mousa S. A. (2016). Thymoquinone modulates blood coagulation in vitro via its effects on inflammatory and coagulation pathways. *Intern. J. Mol. Sci.* 17:474. 10.3390/ijms17040474 27043539 PMC4848930

[B31] Ondresik M. Azevedo Maia F. R. da Silva Morais A. Gertrudes A. C. Dias Bacelar A. H. Correia C. (2017). Management of knee osteoarthritis. Current status and future trends. *Biotechnol. Bioeng.* 114 717–739. 10.1002/bit.26182 27618194

[B32] Quinn R. H. Murray J. N. Pezold R. Sevarino K. S. (2018). Surgical management of osteoarthritis of the knee. *J. Am. Acad. Orthop. Surg.* 26 e191–e193. 10.5435/JAAOS-D-17-00424 29688919

[B33] Ramalingam M. Kim H. Lee Y. Lee Y.-I. (2018). Phytochemical and pharmacological role of liquiritigenin and isoliquiritigenin from radix glycyrrhizae in human health and disease models. *Front. Aging Neurosci.* 10:348. 10.3389/fnagi.2018.00348 30443212 PMC6221911

[B34] Rojo A. I. McBean G. Cindric M. Egea J. Lopez M. G. Rada P. (2014). Redox control of microglial function: molecular mechanisms and functional significance. *Antioxid. Redox Signal.* 21 1766–1801. 10.1089/ars.2013.5745 24597893 PMC4186766

[B35] Sanphui P. Pramanik S. K. Chatterjee N. Moorthi P. Banerji B. Biswas S. C. (2013). Efficacy of cyclin dependent kinase 4 inhibitors as potent neuroprotective agents against insults relevant to Alzheimer’s disease. *PLoS One* 8:e78842. 10.1371/journal.pone.0078842 24244372 PMC3823981

[B36] Sassi N. Laadhar L. Allouche M. Achek A. Kallel-Sellami M. Makni S. (2014). WNT signaling and chondrocytes: from cell fate determination to osteoarthritis physiopathology. *J. Recept. Signal Transd.* 34 73–80. 10.3109/10799893.2013.863919 24303940

[B37] Scanzello C. R. (2017). Chemokines and inflammation in osteoarthritis: insights from patients and animal models. *J. Orthopaed. Res.* 35 735–739. 10.1002/jor.23471 27808445 PMC5912941

[B38] Shen J. Abu-Amer Y. O’Keefe R. J. McAlinden A. (2017). Inflammation and epigenetic regulation in osteoarthritis. *Connect. Tissue Res.* 58 49–63. 10.1080/03008207.2016.1208655 27389927 PMC5266560

[B39] Shuid A. N. Mohamed N. Mohamed I. N. Othman F. Suhaimi F. Mohd Ramli E. S. (2012). Nigella sativa: a potential antiosteoporotic agent. *Evid. Based Complement. Altern. Med.* 2012:696230. 10.1155/2012/696230 22973403 PMC3438907

[B40] Stocco E. Barbon S. Petrelli L. Piccione M. Belluzzi E. Pozzuoli A. (2019). Infrapatellar fat pad stem cells responsiveness to microenvironment in osteoarthritis: from morphology to function. *Front. Cell Dev. Biol.* 7:323. 10.3389/fcell.2019.00323 31921840 PMC6914674

[B41] Tabeshpour J. Mehri S. Abnous K. Hosseinzadeh H. (2019). Role of oxidative stress, MAPKinase and apoptosis pathways in the protective effects of thymoquinone against acrylamide-induced central nervous system toxicity in rat. *Neurochem. Res.* 45 254–267. 10.1007/s11064-019-02908-z 31728856

[B42] Tarailo-Graovac M. Shyr C. Ross C. J. Horvath G. A. Salvarinova R. Ye X. C. (2016). Exome sequencing and the management of neurometabolic disorders. *New Engl. J. Med.* 374 2246–2255. 10.1056/NEJMoa1515792 27276562 PMC4983272

[B43] Tian J. Shi J. Zhang X. Wang Y. (2010). Herbal therapy: a new pathway for the treatment of Alzheimer’s disease. *Alzheimer Res. Therap.* 2:30. 10.1186/alzrt54 21067555 PMC2983439

[B44] Umar S. Hedaya O. Singh A. K. Ahmed S. (2015). Thymoquinone inhibits TNF-α-induced inflammation and cell adhesion in rheumatoid arthritis synovial fibroblasts by ASK1 regulation. *Toxicol. Appl. Pharmacol.* 287 299–305. 10.1016/j.taap.2015.06.017 26134265 PMC4549173

[B45] Velagapudi R. El-Bakoush A. Lepiarz I. Ogunrinade F. Olajide O. A. (2017). AMPK and SIRT1 activation contribute to inhibition of neuroinflammation by thymoquinone in BV2 microglia. *Mol. Cell. Biochem.* 435 149–162. 10.1007/s11010-017-3064-3 28551846 PMC5632349

[B46] Vizoso F. J. Eiro N. Cid S. Schneider J. Perez-Fernandez R. (2017). Mesenchymal stem cell secretome: toward cell-free therapeutic strategies in regenerative medicine. *Intern. J. Mol. Sci.* 18:1852. 10.3390/ijms18091852 28841158 PMC5618501

[B47] Wang C. (2013). Complementary and alternative medicine and osteoarthritis. *Intern. J. Integr. Med.* 1:13. 10.5772/56431 28835780 PMC5565213

[B48] Wang D. Qiao J. Zhao X. Chen T. Guan D. (2015). Thymoquinone inhibits IL-1β-induced inflammation in human osteoarthritis chondrocytes by suppressing NF-κB and MAPKs signaling pathway. *Inflammation* 38 2235–2241. 10.1007/s10753-015-0206-1 26156811

[B49] Wang M. Shen J. Jin H. Im H.-J. Sandy J. Chen D. (2011). Recent progress in understanding molecular mechanisms of cartilage degeneration during osteoarthritis. *Ann. N. Y. Acad. Sci.* 1240:61. 10.1111/j.1749-6632.2011.06258.x 22172041 PMC3671949

[B50] Xu P. Huang M.-W. Xiao C.-X. Long F. Wang Y. Liu S.-Y. (2017). Matairesinol suppresses neuroinflammation and migration associated with Src and ERK1/2-NF-κB pathway in activating BV2 microglia. *Neurochem. Res.* 42 2850–2860. 10.1007/s11064-017-2301-1 28512713

[B51] Youssef P. Chami B. Lim J. Middleton T. Sutherland G. T. Witting P. K. (2018). Evidence supporting oxidative stress in a moderately affected area of the brain in Alzheimer’s disease. *Sci. Rep.* 8:11553. 10.1038/s41598-018-29770-3 30068908 PMC6070512

